# SPARC modulates macrophage-like phenotype transformation in VSMCs and triggers a vicious cycle

**DOI:** 10.3389/fcell.2026.1791698

**Published:** 2026-05-07

**Authors:** Gengfan Ye, Hongcai Wang, Kuan Feng, Shiwei Li, Xuebin Wen, Zhenqiang Li, Wei Chen, Maosong Chen

**Affiliations:** 1 Department of Neurosurgery, The Affiliated Lihuili Hospital of Ningbo University, Ningbo, Zhejiang, China; 2 Department of Anesthesiology, The Affiliated Lihuili Hospital of Ningbo University, Ningbo, Zhejiang, China

**Keywords:** intracranial aneurysm, SPARC, VSMCs, macrophage-like phenotype transformation, ROS, aspirin

## Abstract

Intracranial aneurysms (IAs) are characterized by abnormal cerebral artery dilations and pose significant risks due to their potential for rupture, leading to subarachnoid hemorrhage. Although inflammation and vascular smooth muscle cell (VSMC) phenotypic transformations are implicated in IA pathogenesis, the role of secreted protein acidic and rich in cysteine (SPARC) in these processes remains poorly understood. This study explored the role of SPARC in inducing macrophage-like phenotypic transformation of VSMCs using data from the Gene Expression Omnibus (GEO) database and *in vitro* experiments. SPARC overexpression in VSMCs was induced, and its effects on VSMC phenotype markers, reactive oxygen species (ROS) production, macrophage chemotaxis, and lipid accumulation were evaluated. Additionally, molecular docking was used to explore the potential interaction between SPARC and aspirin. SPARC overexpression induced a shift from contractile to macrophage-like and pro-inflammatory VSMC phenotypes, which was associated with elevated ROS production. ROS inhibition blocked this transformation. SPARC also upregulated markers related to macrophage-like behavior and enhanced VSMC migration. Furthermore, molecular docking revealed potential binding of aspirin to SPARC, and aspirin treatment mitigated macrophage-like VSMC transformation in a TNF-α-induced IA model. SPARC promotes macrophage-like transformation in VSMCs through ROS production, contributing to IA pathogenesis. Targeting SPARC or ROS may offer therapeutic strategies for preventing IA rupture. Aspirin’s potential to regulate SPARC expression opens new avenues for IA treatment.

## Introduction

Intracranial aneurysms (IAs) are localized pathological dilations of the cerebral artery, characterized by weak and abnormal vessel walls (Frosen et al., 2012). Established risk factors for IAs include genetic susceptibility, race, advanced age, and female gender (Juvela, 2019). Genetic studies have also identified modifiable risk factors such as blood pressure, smoking, educational attainment, and insomnia (Sun et al., 2022). Recent research has highlighted an association between gut microbiome dysbiosis and IA formation (Kawabata et al., 2022). The overall prevalence of intracranial aneurysms in the adult population is estimated to be approximately 2%–3%, based on population-based imaging and epidemiological studies (Vlak et al., 2011). Meanwhile, ongoing research continues to identify additional risk factors associated with IA formation and progression.

While most patients with unruptured IAs remain asymptomatic throughout their lives, they still face a rupture risk ranging from 0.5% to 1.0% per aneurysm year (Juvela, 2021). A ruptured IA can lead to aneurysmal subarachnoid hemorrhage (aSAH), which carries a high rate of morbidity and mortality (Long et al., 2017). Despite advances in neurosurgical intensive care, the mortality rate for aSAH remains between 30% and 40% (Nieuwkamp et al., 2009), and nearly half of the survivors experience disabilities or long-term cognitive impairment (Neifert et al., 2021).

Although microsurgical clipping and endovascular therapy are effective in preventing IA rupture, both are associated with procedural risks (Naggara et al., 2012; Kotowski et al., 2013). Notably, there are currently no well-established pharmacological therapies for IAs, highlighting the need for further investigation into their underlying pathogenesis to identify novel therapeutic targets.

The exact mechanisms underlying the occurrence, development, and rupture of IAs remain poorly understood. However, inflammation infiltration and aneurysm wall remodeling are considered key pathological features in IAs (Liu et al., 2022). Vascular smooth muscle cells (VSMCs) play a crucial role in mediating vascular remodeling, and their phenotypic characteristics can serve as valuable indicators for the early identification, staging, and rupture risk prediction of IAs (Li and Wang, 2019). Numerous molecules have been identified as participants in the development of IAs by affecting VSMC function (Wang et al., 2022).

Our previous study highlighted the role of secreted protein acidic and rich in cysteine (SPARC) in maintaining smooth muscle cell homeostasis (Ye et al., 2016). SPARC, also known as BM-40, is located on chromosome 5q31.3-5q32. It is secreted by endothelial cells, VSMCs, osteoblasts, and other cell types, and plays a key role in regulating extracellular matrix (ECM) remodeling, cell adhesion, and the cell cycle (Jiang et al., 2023). We found that SPARC is highly expressed in IA tissues and may be a reliable predictor of IA rupture. Predicting IA rupture using SPARC yields a significant clinical net benefit. Our preliminary investigations suggest that SPARC could be a potential pathogenic target for IAs. Previous studies have indicated that SPARC is widely expressed in IA tissues and may contribute to ECM remodeling during aneurysm formation (Li et al., 2013). More recent transcriptomic and molecular analyses further support the upregulation of SPARC in aneurysm tissues and its involvement in vascular wall degeneration, ECM degradation, and inflammatory remodeling processes (Kumar et al., 2024).

Regarding VSMC phenotypic characteristics, it has been reported that SPARC can induce the transformation of contractile VSMCs to inflammatory (Tan et al., 2020) and synthetic (Li et al., 2019) phenotypes. Under pathological conditions, contractile VSMCs can transform into various non-contractile phenotypes, including dedifferentiated, synthetic, pro-inflammatory, macrophage-like, foam-cell, and osteoblast-like VSMCs (Wang et al., 2023). However, there is limited research examining the relationship between SPARC and phenotypic transformations beyond the pro-inflammatory and synthetic phenotypes.

## Methods

### Data mining

In this study, we analyzed four types of VSMC phenotypic biomarkers: synthetic, dedifferentiated, pro-inflammatory, and macrophage-like.

Synthetic markers: Cyclin B1 (CCNB1), DNA topoisomerase 2-alpha (TOP2A), minichromosome maintenance complex components 2 and 3 (MCM2, MCM3), replication factor C subunit 2 (RFC2), and hepatocellular carcinoma-associated protein 2 (HCCA2). It should be noted that several of these markers (e.g., CCNB1, TOP2A, MCM2, and MCM3) are classical regulators of cell cycle progression and proliferation. In the context of VSMC biology, they are commonly used as indirect indicators of the synthetic phenotype due to its association with increased proliferative activity; however, they may not represent phenotype-specific markers *per se*.

Dedifferentiated markers: Activating Transcription Factor 3 (ATF-3), Transcription Factor Jun (JUN), and Protein C-Fos (FOS).

Pro-inflammatory markers: C-X-C Motif Chemokine Ligand 2 (CXCL2), C-C Motif Chemokine Receptor Like 2 (CCRL2), Matrix Metallopeptidases 9 and 13 (MMP9, MMP13), C-C Motif Chemokine Ligand 2 (CCL2), Tumor Necrosis Factor (TNF), Interleukin 6 (IL-6), and IL-8.

Macrophage-like markers: Galectin 3 (LGALS3), CD68 Molecule (CD68), Complement C3a Receptor 1 (C3AR1), Integrin Subunit Alpha X (ITGAX), and ITGAM.

To identify clinical correlations, we retrieved IA data from the Gene Expression Omnibus (GEO) database (https://www.ncbi.nlm.nih.gov/geo/). Regarding the selection criteria in GEO, Entry type was Series, Study type was Expression profiling by array, Organisms was *Homo sapiens*, and samples in the dataset included intracranial aneurysms and its paired samples. Finally, the GSE75436 dataset (based on the GPL10558 platform) was selected, which includes 15 IA samples and 15 matched control samples. First, we examined the differential expression of VSMC phenotype biomarkers between control and IA groups by Wilcoxon signed-rank test. Next, we analyzed the correlation between SPARC and various VSMC phenotypic biomarkers in both groups by Spearman method. Given this strong correlation, our subsequent investigations focused on validating the role of SPARC in driving macrophage-like phenotypic transformation in VSMCs.

### Materials

Human brain VSMCs (Catalog# FH1244) and human bone marrow-derived macrophages (Catalog# FH-H096) were obtained from FuHeng Biology, China. THP-1 human monocytic cells were obtained from Shanghai Jinyuan Biotechnology Co., Ltd. (Shanghai, China). Antibodies against Alpha-Smooth Muscle Actin (α-SMA, Catalog# ET1607-53), SPARC (Catalog# ER1916-99), Osteopontin (OPN, Catalog# 0806-6), Galectin 3 (LGALS3, Catalog# ER1912-28), CD68 Molecule (CD68, Catalog# ER1901-32), Interleukin 6 (IL-6, Catalog# R1412-2), Matrix Metallopeptidase 9 (MMP9, Catalog# ET1704-69), MMP13 (Catalog# ET1702-14), TIMP Metallopeptidase Inhibitor 4 (TIMP4, Catalog# ER65170), Collagen-II (Catalog# ER1906-49), E-cadherin (Catalog# ET1607-75), N-cadherin (Catalog# ET1607-37), Vimentin (Catalog# ET1610-39), Monocyte Chemoattractant Protein-1 (MCP-1, Catalog# ET1611-65), Integrin Subunit Alpha X (ITGAX, Catalog# RT1108), ITGAM (Catalog# RT1107), Tumor Necrosis Factor (TNF, Catalog# ER65189), and, Kruppel-Like Factor 4 (KLF4, Catalog# ET1702-71) were from the HuaBio, China. Smooth Muscle Protein 22-Alpha (SM22-α, Catalog# 10493-1-AP) was from Proteintech. The kits for reactive oxygen species (ROS) detection (Catalog# CA1410) and oil red O staining (Catalog# G1262) were from Solarbio Life Science, China. The ROS inhibitor acetylcysteine (Catalog# HY-B0215), aspirin (Catalog# HY-14654), and bindarit (Catalog# HY-B0498) were from the MedChemExpress, China.

### Cell culture and transfection

VSMCs were cultured in the recommended smooth muscle cell culture medium (ScienCell) at 37 °C under 95% air and 5% CO_2_. For this study, VSMCs were passaged 3-5 times prior to experiments. To induce overexpression of SPARC, a recombinant plasmid containing the full-length human SPARC coding sequence (NM_003118.4) was constructed by Genecreate Biological Engineering Co., Ltd. (Wuhan, China). The SPARC coding sequence was synthesized and subcloned into a mammalian expression vector by the manufacturer. The construct was verified by Sanger sequencing. The following primers provided by the manufacturer were used for verification of the SPARC insert: Forward (F): 5′-GGC​GGC​GTG​TGG​CAG​GAG-3'; Reverse (R): 5′-CCA​GAG​AAG​AGG​TAA​CAA​CGA​GAG​AT-3'. VSMCs were transfected with the SPARC overexpression plasmid or the corresponding empty vector control using Lipofectamine 3000 transfection reagent (Invitrogen, Catalog# L3000015) according to the manufacturer’s instructions. Briefly, 2.5 μg of plasmid DNA per well (6-well plate) was mixed with Lipofectamine 3000 and P3000 reagent in Opti-MEM reduced serum medium (Gibco, Catalog# 31985062) and added to cells at approximately 70%–80% confluence. After 48 h of transfection, total proteins from the VSMCs were extracted. The protein levels of SPARC were measured by Western blot to assess the transfection efficiency.

### The detection of macrophage-like biomarkers and ROS content in VSMCs

VSMCs were treated to induce high expression of SPARC. Western blot analysis was then performed to assess the expression levels of phenotypic biomarkers including α-SMA, SM22-α, OPN, LGALS3, and CD68.

For ROS detection, SPARC-overexpressing VSMCs were harvested, resuspended, and adjusted to a concentration of 1 × 10^6^ cells/mL. Following phosphate-buffered saline (PBS) washing, DCFH-DA (2′,7′-dichlorofluorescein diacetate) was added to the cell suspension to achieve a final concentration of 10 μmol/L. The cells were then incubated at 37 °C for 30 min in the dark. Subsequently, the cell suspension was gently mixed to ensure complete loading of the fluorescent probe. After incubation, the cells were washed three times with PBS to remove any extracellular DCFH-DA that had not penetrated into the cells. Within 1 hour of staining, fluorescence intensity was measured using flow cytometry (excitation wavelength 485 nm, emission wavelength 530 nm), which directly reflected the intracellular ROS levels.

### Quantitative real-time PCR (RT-qPCR)

RT-qPCR was performed to evaluate the mRNA expression changes of ROS generation-related genes following SPARC overexpression. Total RNA was extracted from VSMCs using TRIzol reagent (Invitrogen, Catalog# 15596026) according to the manufacturer’s instructions. RNA purity and concentration were assessed using a NanoDrop 2000 spectrophotometer (Thermo Fisher Scientific), and samples with an A260/A280 ratio between 1.8 and 2.0 were used for subsequent experiments. Complementary DNA (cDNA) was synthesized from 1 μg of total RNA using the PrimeScript RT Reagent Kit with gDNA Eraser (Takara, Catalog# RR047A) following the manufacturer’s protocol. Quantitative real-time PCR was then performed using TB Green Premix Ex Taq II (Takara, Catalog# RR820A) on an Applied Biosystems QuantStudio 5 Real-Time PCR System (Thermo Fisher Scientific). Three ROS generation-related genes were examined: NADPH Oxidase 4 (NOX4), Superoxide Dismutase 1 (SOD1), and Nuclear Respiratory Factor 1 (NRF1). The thermal cycling conditions were as follows: initial denaturation at 95 °C for 1 min, followed by 40 cycles of 95 °C for 5 s and 60 °C for 20 s. Glyceraldehyde-3-phosphate dehydrogenase (GAPDH) was used as the internal reference gene for normalization. Relative mRNA expression levels were calculated using the 2^−ΔΔCT^ method. All reactions were performed in triplicate. The following primers were used:

NOX4, F: 5′-CTC​AGC​GGA​ATC​AAT​CAG​CTG​TG-3′

NOX4, R: 5′-AGA​GGA​ACA​CGA​CAA​TCA​GCC​TTA​G-3′

SOD1, F: 5′-CAT​CAG​CCC​TAA​TCC​ATC​TGA-3′

SOD1, R: 5′-CGC​GAC​TAA​CAA​TCA​AAG​TGA-3′

NRF1, F: 5′-TGG​AAC​AGC​AGT​GGC​AAG​ATC​TCA-3′

NRF1, R: 5′-GGC​ACT​GTA​CAG​GAT​TTC​ACT​TGC-3′

GAPDH, F: 5′-GTC​TCC​TCT​GAC​TTC​AAC​AGC​G-3′

GAPDH, R: 5′-ACC​ACC​CTG​TTG​CTG​TAG​CCA​A-3′

In addition, we explored the correlation between SPARC and mitochondria function related biomarkers in matched control and IAs samples using GSE75436 dataset, respectively. Mitochondria function related biomarkers contained mitochondrial oxidative phosphorylation biomarkers (NADH: Ubiquinone Oxidoreductase Subunit B8, NDUFB8), mitochondrial biogenesis biomarkers (Progastricsin, PGC; NRF1; Transcription Factor a Mitochondrial, TFAM), mitochondrial fusion biomarkers (Mitofusin 1, MFN1; MFN2; Mitochondrial Dynamin Like GTPase, OPA1), and mitochondrial fission biomarkers (Dynamin 1 Like, DNM1L; Fission, Mitochondrial 1, FIS1; Mitochondrial Fission Factor, MFF). We also explored the correlation between SPARC and mitochondrial DNA (mtDNA) encoded proteins (Cytochrome C Oxidase Subunit I, COX1; COX2; COX3; ATP6).

### Western blot

Protein extraction was performed using RIPA lysis buffer (Beyotime, Catalog# P0013B) supplemented with protease inhibitor cocktail (Beyotime, Catalog# P1005). Protein concentrations were determined using a BCA Protein Assay Kit (Beyotime, Catalog# P0012S). Equal amounts of protein (20–30 μg per lane) were separated by 10%–12% SDS-PAGE and transferred onto PVDF membranes (Millipore, Catalog# IPVH00010). After blocking with 5% non-fat milk in TBST, the membranes were incubated overnight at 4 °C with primary antibodies targeting IL-6, MMP9, MMP13, TIMP4, Collagen-II, MCP-1, ITGAX, ITGAM, α-SMA, SM22-α, OPN, LGALS3, and CD68. The primary antibodies were used at optimal dilutions as follows: IL-6 (1:1000), MMP9 (1:500), MMP13 (1:500), TIMP4 (1:1000), Collagen-II (1:1000), MCP-1 (1:1000), ITGAX (1:1000), ITGAM (1:1000), α-SMA (1:1000), SM22-α (1:1500), OPN (1:800), LGALS3 (1:1000), CD68 (1:1200), and GAPDH (1:5000, Proteintech, Catalog# 60004-1-Ig). GAPDH was used as the loading control for normalization of all Western blot experiments. After washing three times with TBST (10 min each), membranes were incubated with HRP-conjugated goat anti-rabbit IgG secondary antibody (HuaBio, Catalog# HA1001, 1:5000) or HRP-conjugated goat anti-mouse IgG secondary antibody (HuaBio, Catalog# HA1006, 1:5000) for 1 h at room temperature. Protein bands were detected using an enhanced chemiluminescence (ECL) kit (Beyotime, Catalog# P0018S) and visualized with a chemiluminescence imaging system (Bio-Rad ChemiDoc XRS+). Densitometric analysis of protein bands was performed using ImageJ software (National Institutes of Health, version 1.53). The relative expression levels of target proteins were normalized to GAPDH, and fold changes were calculated relative to the control group.

### Wound healing assay

To assess VSMC migration, a wound healing assay was performed. SPARC-overexpressing VSMCs (5 × 10^5^ cells/well) were plated in a 6-well plate and cultured under standard conditions (37 °C, 5% CO_2_). A 96-well replicator (VP Scientific) was used to create a scratch on the cell monolayer. The scratched area was cleared of detached cells, and the cells were incubated for 24 h. Migration rates were determined by photographing the wound area and calculating the distance covered by the cells.

### THP-1 differentiation and co-culture system with VSMCs

THP-1 human monocytic cells (ATCC, TIB-202) were cultured in RPMI 1640 medium (Gibco) supplemented with 10% fetal bovine serum (FBS) and 1% penicillin-streptomycin. To differentiate THP-1 monocytes into macrophages, cells were treated with 100 ng/mL phorbol 12-myristate 13-acetate (PMA; Sigma-Aldrich, Catalog# P1585) for 48 h, followed by a 24-h resting period in PMA-free medium to allow full differentiation into adherent macrophage-like cells. Differentiation was confirmed by cell morphology (adherent, spread morphology) and upregulation of the macrophage marker CD68 by Western blot. The PMA-differentiated THP-1 macrophages were then used for co-culture experiments. A co-culture system was established using Transwell inserts with 0.4 μm pore membranes (Merck Millipore, United States), which allow paracrine signaling via soluble factors while preventing direct cell–cell contact and cell migration. VSMCs were plated on the bottom of a 6-well cell culture plate, and PMA-differentiated THP-1 macrophages were seeded on the upper Transwell membrane. Experiments were performed after overnight culture, and VSMCs were collected separately for further analysis.

### Transwell migration assay

To evaluate macrophage chemotaxis toward SPARC-overexpressing VSMCs, a Transwell migration assay was performed using 24-well Transwell inserts with 8 μm pore polycarbonate membranes (Corning, Catalog# 3422), which permit cell migration through the membrane. PMA-differentiated THP-1 macrophages (1 × 105 cells/well) were resuspended in serum-free medium and seeded in the upper chamber of the Transwell insert, while the lower chamber contained medium supplemented with 30% FBS (600 μL/well). After 24 h of incubation at 37 °C with 5% CO_2_, non-migratory cells remaining on the upper surface of the membrane were removed by gently wiping with a cotton swab. The cells that had migrated to the lower surface of the membrane were fixed with 4% paraformaldehyde for 20 min, stained with 0.1% crystal violet solution for 15 min, and counted under a light microscope. Five random fields per membrane were counted at ×200 magnification to assess migration.

### Oil red O staining for lipid accumulation

To examine lipid accumulation in the VSMCs, cells were cultured in 24-well sterile culture plates. The cells were gently rinsed twice with PBS solution and fixed with 4% paraformaldehyde for 30 min. The PBS solution was rinsed for 1 min, oil red O working fluid was applied in an oven at 60 °C for 15–20 min, and the cells were washed with distilled water 1–2 times, 1–2 min each time. Subsequently, 60% isopropanol was separated and the red intracellular lipid droplets were observed under the microscope. The images were collected and the experimental results were analyzed by ImageJ analysis software. The intracellular lipid droplets were expressed by integrated optical density.

### ROS inhibition treatment

To explore the role of ROS in SPARC-induced phenotypic changes, VSMCs overexpressing SPARC were treated with a 6 mM ROS inhibitor (acetylcysteine). Protein expression of IL-6, MMP9, MMP13, Collagen-II, and other markers, as well as macrophage chemotaxis and lipid accumulation, were re-evaluated after the ROS inhibition treatment.

### Study of binding interaction

The binding interaction between aspirin and SPARC was initially investigated using molecular docking analysis. The protein structure of SPARC was obtained from the Protein Data Bank (PDB), and the SDF structure of aspirin was retrieved from the PubChem database. Molecular docking analysis was performed using AutoDockVina1.1.2 software to explore the potential binding of aspirin to SPARC.

To induce the phenotypic switch in VSMCs, TNF-α (100 ng/mL) treatment was applied for 24 h, which is commonly used to model IA cell phenotypic changes (Sun et al., 2023). Aspirin (1 mM) treatment was then applied to the IA cell model exhibiting high SPARC expression for an additional 24 h.

### Statistical analysis

Statistical analysis was performed using SPSS 23.0. The correlation between two variables was assessed using the Spearman method. The differences in mRNA expression between two groups were compared using a t-test, assuming the data followed a normal distribution. For comparisons among three or more groups, one-way analysis of variance (ANOVA) followed by Tukey’s post hoc test was used. Data are presented as mean ± standard deviation (SD). All experimental results in this study were obtained from at least three independent replicates. A p-value of <0.05 was considered statistically significant.

## Results

### Identification of potential biomarkers correlated with SPARC

**FIGURE 1:**
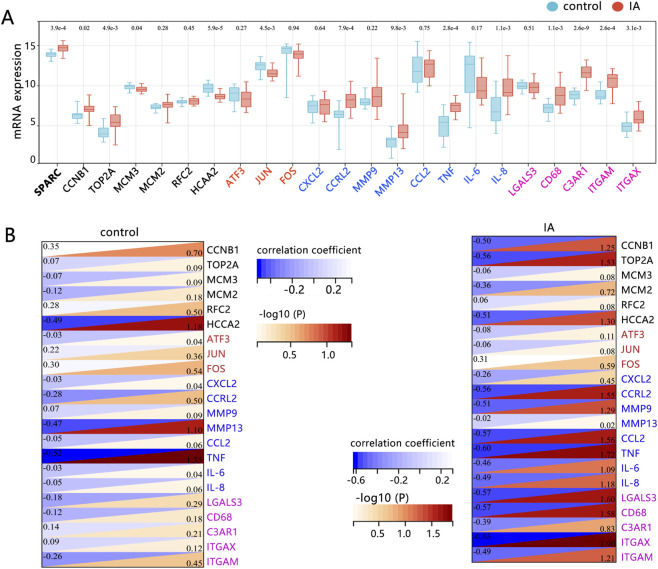
Identification of Potential Biomarkers Correlated with SPARC. **(A)** Expression differences of various phenotypic biomarkers between the matched control and IAs groups. Statistical significance: *P < 0.05, **P < 0.01, ***P < 0.001. **(B)** Correlation analysis between SPARC and different phenotypic biomarkers of VSMCs in both control and IAs groups, based on the GSE75436 dataset (n = 15 in each group). The black (excluding SPARC), red, blue, and rose-red colors represent synthetic, dedifferentiated, pro-inflammatory, and macrophage-like VSMC biomarkers, respectively.

We initially observed that SPARC expression was significantly elevated in the IAs group compared to the control group (Figure 1A). Further analysis of four phenotypic biomarkers revealed similar trends to SPARC, with several pro-inflammatory and macrophage-like VSMC markers, such as CCRL2 and MMP13, showing marked overexpression in the IAs group. We then assessed the correlation between SPARC and various VSMC phenotypic biomarkers. In the control group, only TNF showed a significant correlation with SPARC (Figure 1B). In contrast, in the IAs group, SPARC exhibited a strong correlation with macrophage-like biomarkers, while pro-inflammatory biomarkers showed a secondary correlation.

### The effect of SPARC on macrophage-like phenotype transformation of VSMCs and oxidative stress

**FIGURE 2:**
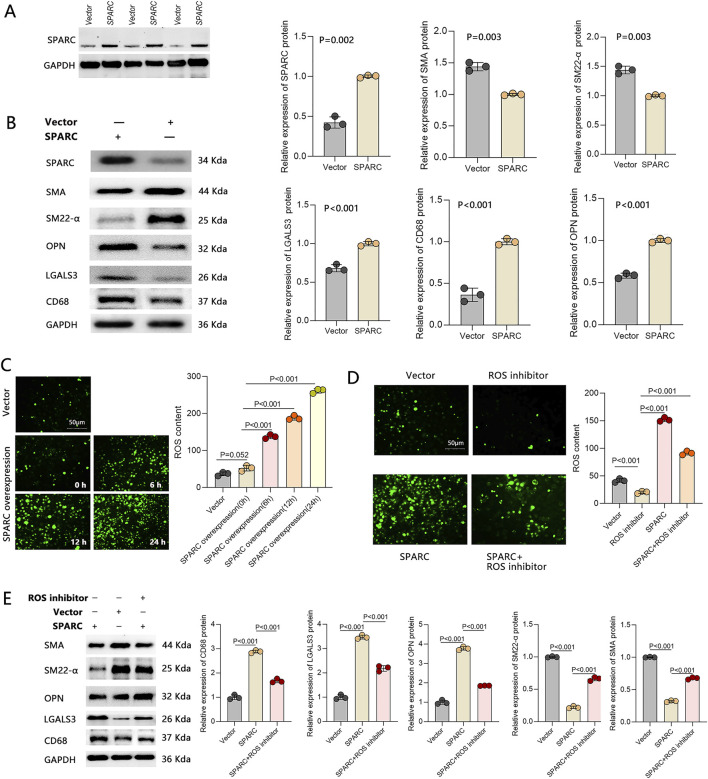
The Effect of SPARC on Macrophage-like Phenotype Transformation of VSMCs and Oxidative Stress. VSMCs were treated with SPARC overexpression, and **(A)** transfection efficiency detection, **(B)** the expression of macrophage-like VSMC biomarkers after transfection, and **(C)** ROS content were measured. Further, VSMCs with SPARC overexpression were treated with a ROS inhibitor, and **(D)** ROS content, along with **(E)** the expression of macrophage-like biomarkers, were reassessed (n = 3 in each group).

First, we performed SPARC overexpression in VSMCs and confirmed the successful transfection of the overexpression construct (Figure 2A). We then examined the expression of macrophage-like biomarkers of VSMCs and found that SPARC overexpression led to a downregulation of contractile markers (α-SMA and SM22-α) and an upregulation of synthetic biomarkers like OPN. Additionally, macrophage-like markers, including LGALS3 and CD68, were also increased (Figure 2B), supporting the idea that SPARC overexpression promotes macrophage-like phenotype transformation in VSMCs.

Previous studies have shown that SPARC upregulation in VSMCs induces oxidative stress and increases ROS production, which triggers phenotype transformation in contractile VSMCs (Sun et al., 2023). We hypothesized that the macrophage-like phenotype transformation induced by SPARC might also be linked to ROS production. To test this, we measured ROS levels via immunofluorescence and found a significant, time-dependent increase in ROS upon SPARC overexpression (Figure 2C). Using a ROS inhibitor effectively prevented the SPARC-induced ROS increase (Figure 2D). Importantly, ROS inhibition resulted in the upregulation of SM22-α and the downregulation of LGALS3 and CD68 (Figure 2E), suggesting that blocking ROS production can prevent the macrophage-like phenotype transformation of VSMCs induced by SPARC. Our results indicate that SPARC-induced macrophage-like transformation in VSMCs is closely associated with elevated ROS production.

### The effect of SPARC on enzymatic activity related to ROS generation

**FIGURE 3:**
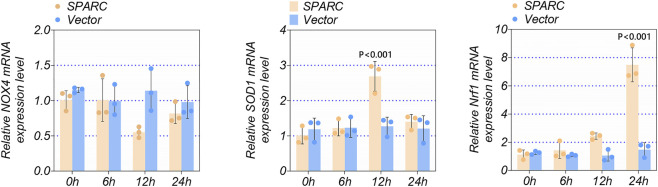
The Effect of SPARC on Enzymatic Activity Related to ROS Generation. The activity of enzymes involved in ROS production was assessed following SPARC overexpression (n = 3 in each group). **P < 0.01.

ROS production is regulated by multiple factors. To further elucidate SPARC’s role, we examined three key biomarkers involved in ROS generation: NOX4, SOD1, and NRF1. Our results showed that SPARC overexpression significantly upregulated NRF1 expression (Figure 3, P < 0.01), suggesting a potential link between SPARC and oxidative stress regulation.

### Correlation analysis between SPARC and mitochondrial function-related biomarkers

**FIGURE 4:**
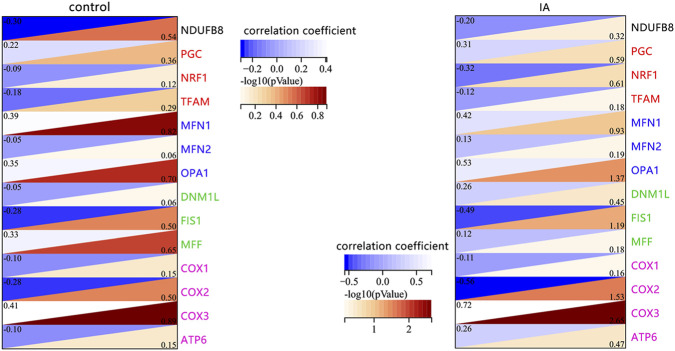
Correlation Analysis Between SPARC and Mitochondrial Function-Related Biomarkers in Control and IAs Patients. Biomarkers related to mitochondrial oxidative phosphorylation (black), biogenesis (red), fusion (blue), fission (green), and mtDNA-encoded proteins (rose-red) were analyzed. Data from the GSE75436 dataset (n = 15 in each group).

However, no significant correlation was found between SPARC and mitochondrial biogenesis biomarkers (PGC-1α, TFAM) in either control or IAs patients (Figure 4). To further investigate, we explored the relationship between SPARC and oxidative phosphorylation (OXPHOS) biomarkers, but again, no significant correlation was observed between SPARC and the OXPHOS-related biomarker (NDUFB8) in either group (Figure 4). Interestingly, we did find a strong correlation between SPARC and mitochondrial DNA (mtDNA)-encoded OXPHOS biomarkers (COX2, COX3, all P < 0.05).

### SPARC overexpression induces a vicious cycle of macrophage-like VSMCs

**FIGURE 5:**
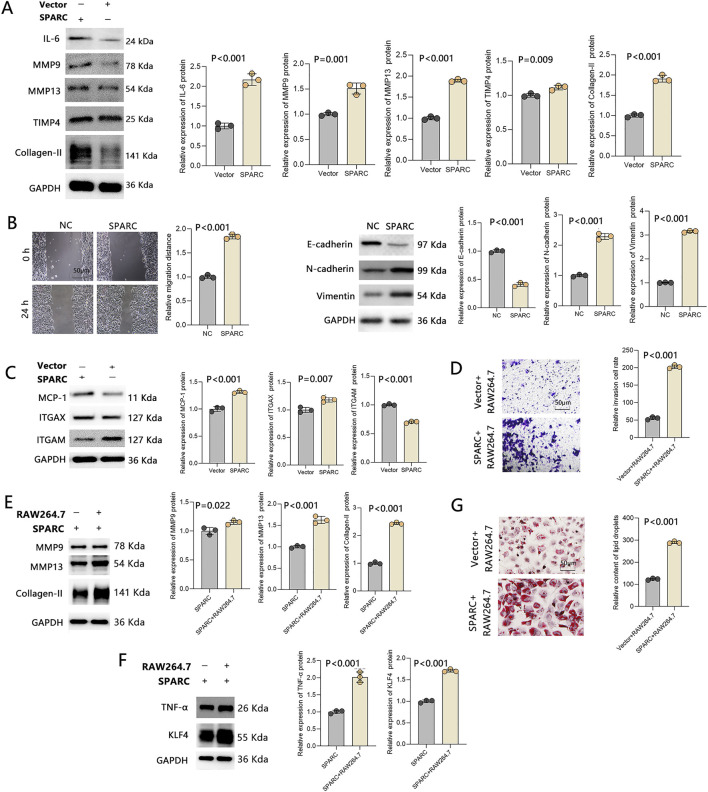
SPARC Overexpression Induces a Vicious Cycle of Macrophage-Like VSMCs. **(A)** Expression of biomarkers secreted by macrophage-like VSMCs. **(B)** Migration ability of macrophage-like VSMCs. **(C)** Expression of biomarkers linked to macrophage infiltration. **(D)** Chemotactic activity of macrophages induced by macrophage-like VSMCs. **(E)** Macrophage biomarker expression in co-cultured VSMCs and macrophages. **(F)** Biomarkers associated with lipid accumulation. **(G)** Lipid accumulation detected by Oil Red O staining (n = 3 in each group).

The above findings indicate that SPARC overexpression induces macrophage-like phenotype transformation in VSMCs by increasing ROS production, potentially through mitochondrial oxidative phosphorylation and dynamics. To assess the functional consequences, we examined whether SPARC-overexpressing VSMCs exhibited macrophage-like behavior, including biomarker secretion. The results showed a significant increase in IL-6, MMP9, MMP13, and Collagen-II levels (Figure 5A), supporting the acquisition of macrophage-like properties. Given the role of MMPs in cell migration, we also observed enhanced VSMC migration upon SPARC overexpression (Figure 5B).

Additionally, SPARC overexpression reduced ITGAM expression (Figure 5C), facilitating C3 receptor activation, which was accompanied by increased macrophage recruitment (Figure 5D). Co-culture of VSMCs and macrophages led to the upregulation of macrophage biomarkers (Figure 5E) and TNF-α (Figure 5F). Notably, we also detected KLF4 upregulation and lipid accumulation in this group (Figures 5F,G), further reinforcing the link between SPARC expression and macrophage-like transformation in VSMCs.

### Effects of ROS inhibition and macrophage recruitment blockade on the vicious cycle

**FIGURE 6:**
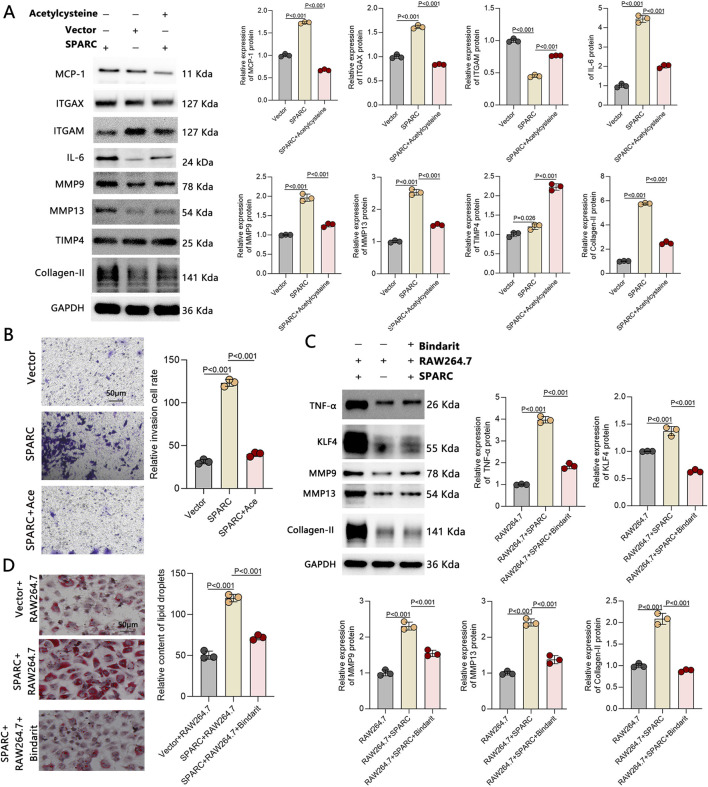
Effects of ROS Inhibition and Macrophage Recruitment Blockade on the Vicious Cycle. **(A)** Expression of macrophage-like biomarkers in VSMCs after ROS inhibition. **(B)** Changes in macrophage chemotactic activity after ROS inhibition. **(C)** Expression of lipid accumulation-associated biomarkers after macrophage recruitment inhibition. **(D)** Lipid accumulation changes following macrophage recruitment inhibition (n = 3 in each group).

To further investigate its impact on the associated vicious cycle, we treated SPARC-overexpressing VSMCs with a ROS inhibitor. The results showed a significant reduction in macrophage biomarker expression (Figure 6A) and macrophage recruitment (Figure 6B). Furthermore, inhibiting macrophage recruitment led to the downregulation of the TNF-α/KLF4 axis (Figure 6C) and a decrease in lipid accumulation (Figure 6D). These findings suggest that ROS inhibition not only prevents the macrophage-like transformation of VSMCs but also disrupts the self-perpetuating cycle driven by macrophage-like VSMCs.

### Aspirin as a therapeutic regulator of SPARC

**FIGURE 7:**
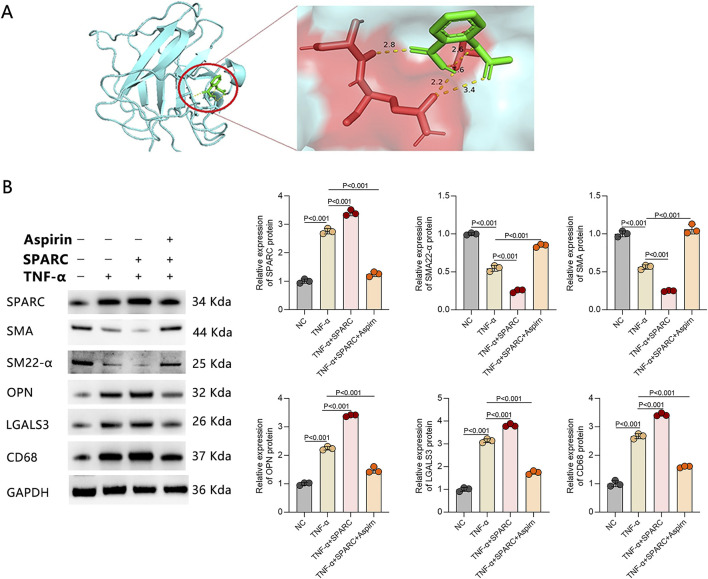
Aspirin as a Therapeutic Regulator of SPARC in IAs. **(A)** Molecular docking analysis demonstrating aspirin’s binding to SPARC. **(B)** Assessment of aspirin’s effects on SPARC expression and macrophage-like VSMCs in an IAs cell model treated with TNF-α (n = 3 in each group).

Our molecular docking analysis demonstrated the successful binding of aspirin to SPARC (Figure 7A). The binding energy between aspirin and SPARC is −4.15 kcal/mol, indicating that the binding is a spontaneous process. Their interaction is primarily driven by van der Waals forces, hydrogen bonds, and desolvation effects, with the combined vdw-hb-desolv-energy being −5.85 kcal/mol. The binding forms a key hydrogen bond, namely, Aspirin (UNL1:H) ↔ SPARC (GLN234:OE1). This hydrogen bond is formed between the aspirin molecule (acting as the hydrogen donor: H) and the side-chain carbonyl oxygen atom (acting as the hydrogen acceptor: OE1) of the glutamine (GLN234) residue at position 234 in the SPARC protein. These results suggest that aspirin may physically bind to the SPARC protein, providing a potential mechanistic hypothesis for some observed non-COX-inhibition-related effects of aspirin. To further investigate, we evaluated the effect of aspirin on SPARC expression and macrophage-like VSMC biomarkers in the established IAs cell model, which was treated with TNF-α. The results showed that TNF-α treatment reduced the expression of normal contractile markers, such as α-SMA and SM22-α, and increased the synthetic biomarker OPN (Figure 7B), confirming the successful establishment of the IAs cell model.

## Discussion

Our previously published study emphasized the crucial role of SPARC in maintaining the homeostasis of VSMCs, suggesting that SPARC may influence the phenotypic characteristics of VSMCs. This study further explored potential biomarkers closely associated with SPARC, specifically focusing on four key phenotypic categories of VSMCs: synthetic, dedifferentiated, pro-inflammatory, and macrophage-like VSMCs. Notably, our results highlight that SPARC may play a central role in modulating the macrophage-like transformation of VSMCs, a process that is pivotal in the pathogenesis of aneurysms.

NRF1, a transcription factor involved in mitochondrial biogenesis and oxidative stress responses, has previously been linked to the regulation of ROS production (Liu et al., 2021a; Liu et al., 2021b). In our study, however, we observed a positive correlation between SPARC overexpression, NRF1 levels, and ROS production. This suggests that SPARC may enhance mitochondrial function through NRF1 activation, which in turn elevates ROS levels. Interestingly, despite NRF1’s role in mitochondrial biogenesis (Gilardini Montani et al., 2019), we found no significant correlation between SPARC and common markers of mitochondrial biogenesis such as PGC-1α or TFAM in either control or IAs patient samples. These findings suggest that the relationship between SPARC and NRF1 may not be directly linked to mitochondrial biogenesis in VSMCs, but rather to other mitochondrial processes such as OXPHOS.

Our results indicate a potential association between SPARC and mitochondrial function, including oxidative phosphorylation and mitochondrial dynamics; however, these observations are primarily correlative and are discussed here in the context of their downstream impact on oxidative stress. Damage to mtDNA has been shown to impair OXPHOS and contribute to excessive ROS production (Qin et al., 2017; Xia et al., 2023). These results suggest that SPARC’s role in mitochondrial dysfunction could be detrimental to the maintenance of the contractile phenotype of VSMCs, which is crucial for vascular stability (Jia et al., 2022). The potential mechanism by which SPARC influences mitochondrial OXPHOS and ROS generation warrants further investigation.

Based on these findings, we propose two non-mutually exclusive mechanistic hypotheses linking SPARC to mitochondrial dysfunction in VSMCs. First, SPARC may influence mitochondrial dynamics, particularly mitochondrial fusion, as suggested by its positive correlation with OPA1, a key regulator of inner mitochondrial membrane fusion. Enhanced mitochondrial fusion has been associated with altered bioenergetics and increased ROS production, which may contribute to phenotypic switching in VSMCs. Second, the observed correlation between SPARC and mtDNA-encoded oxidative phosphorylation proteins (e.g., COX2 and COX3) raises the possibility that SPARC may affect mitochondrial DNA integrity or transcriptional regulation. Disruption of mtDNA integrity can impair oxidative phosphorylation efficiency and lead to excessive ROS generation, thereby promoting cellular stress responses and phenotypic transformation. These mechanisms are not mutually exclusive and may act synergistically, whereby SPARC-induced alterations in mitochondrial dynamics and mtDNA stability collectively contribute to oxidative stress and macrophage-like phenotype transformation in VSMCs. Further experimental studies are required to validate these hypotheses and elucidate the precise molecular pathways involved.

Mitochondrial dynamics, including fusion and fission, have recently been implicated in the regulation of VSMC proliferation, migration, and phenotypic transformation (Xia et al., 2023). Our study evaluated the relationship between SPARC and markers of mitochondrial fusion (MFN1, MFN2, OPA1) and fission (DNM1L, FIS1, MFF). Although no significant correlation was observed in control samples, we found a positive correlation between SPARC and OPA1, a key regulator of mitochondrial fusion, in IAs patients. This suggests that SPARC may promote mitochondrial fusion and ROS generation, which could contribute to the macrophage-like transformation of VSMCs. The specific molecular mechanisms underlying SPARC’s regulation of mitochondrial dynamics and their impact on VSMC phenotype transitions remain to be elucidated.

VSMC migration plays a crucial role in the formation and progression of IAs (Li et al., 2020). It is important to note that VSMCs that express macrophage-like markers are distinct from macrophages, primarily in terms of their antigen processing and presentation capabilities (Vengrenyuk et al., 2015). These macrophage-like VSMCs function as modulators of the inflammatory response, and they secrete biomarkers such as complement component 3 (C3), which binds to receptors like C3AR1, ITGAX, and ITGAM on their surface, promoting macrophage infiltration (Zhang et al., 2024). Our findings support this concept, showing that macrophage recruitment was significantly increased in the presence of SPARC overexpression. Moreover, the upregulation of MCP-1 observed in our study further suggests a link between SPARC-induced macrophage-like VSMCs and macrophage infiltration, which is closely associated with IA development and progression (Aoki et al., 2010; Zhou et al., 2021).

Once recruited, macrophages secrete MMPs that contribute to vascular wall degradation. Additionally, macrophages produce TNF-α, a pro-inflammatory cytokine that accelerates aneurysm formation and rupture (Starke et al., 2014). Our data also show that SPARC-induced macrophage-like VSMCs upregulate TNF-α, which in turn activates the KLF4 pathway, facilitating lipid accumulation and driving further phenotypic modulation of VSMCs (Ji et al., 2019; Zhu et al., 2024). Lipid accumulation, a well-known factor in atherosclerosis, has been implicated in the degeneration and rupture of IA walls (Frosen et al., 2013), and our results suggest that SPARC-mediated macrophage-like VSMC transformation may contribute to this process.

Together, our findings suggest that SPARC overexpression induces the macrophage-like transformation of VSMCs, characterized by upregulation of NRF1, increased ROS production, and enhanced mitochondrial dysfunction. These macrophage-like VSMCs secrete inflammatory biomarkers such as IL-6 and MMPs, which promote migration and contribute to IA progression. Additionally, SPARC-induced macrophage-like VSMCs facilitate macrophage infiltration by activating the C3-ITGAM receptor axis, leading to increased MCP-1 expression and the subsequent recruitment of macrophages. This results in a vicious cycle of inflammation, lipid accumulation, and further phenotype switching of VSMCs. Among the various phenotypic states of VSMCs, the macrophage-like phenotype may be particularly detrimental in the context of intracranial aneurysms. Unlike synthetic or dedifferentiated phenotypes, which primarily contribute to extracellular matrix remodeling and proliferation, macrophage-like VSMCs acquire active pro-inflammatory and immune-like functions. These cells not only lose their contractile properties but also secrete high levels of inflammatory cytokines and matrix-degrading enzymes, such as IL-6 and MMPs, thereby directly contributing to vascular wall degradation.

Furthermore, macrophage-like VSMCs actively promote immune cell recruitment through chemokine secretion and complement-related signaling, amplifying local inflammation. This creates a self-perpetuating cycle in which recruited macrophages further enhance inflammatory signaling, oxidative stress, and phenotypic switching of VSMCs. As a result, this phenotype represents a critical transition from adaptive vascular remodeling to maladaptive inflammatory degeneration, ultimately leading to weakening of the aneurysm wall and increased rupture risk.

It is important to note that some of the biomarkers categorized as synthetic phenotype markers in this study, including CCNB1, TOP2A, MCM2, and MCM3, are primarily associated with cell cycle regulation and cellular proliferation. While the synthetic phenotype of VSMCs is characterized by increased proliferative capacity, these markers may reflect proliferative activity rather than phenotypic switching itself. Therefore, their interpretation should be approached with caution. In contrast, markers associated with macrophage-like and inflammatory phenotypes provide more direct evidence of phenotypic transformation, which represents the primary focus of this study.

Our results suggest that SPARC may serve as a potential therapeutic target in IAs, particularly for modulating macrophage-like phenotype transformation in VSMCs. As such, the development of therapies targeting SPARC could hold significant promise in preventing IA progression. In support of this, the human disease database MalaCards indicates that aspirin (acetylsalicylic acid) is currently approved for the treatment of IAs and is undergoing phase 4 clinical trials. Aspirin has been shown to modulate the inflammatory microenvironment within the IA vessel wall, preventing rupture (Hudson et al., 2019). In the IAs cell model, treatment with aspirin not only inhibited SPARC expression but also reversed the upregulation of macrophage-like biomarkers, such as OPN, LGALS3, and CD86, and restored the expression of normal contractile markers like α-SMA and SM22-α. These findings suggest that aspirin may be an effective agent in preventing the macrophage-like transformation of VSMCs and could represent a promising therapeutic approach for treating IAs.

Our molecular docking analysis demonstrated that aspirin can bind to SPARC, with a binding energy of −4.15 kcal/mol, indicating a relatively weak to moderate interaction. Such binding affinity is generally insufficient for strong competitive inhibition, suggesting that aspirin is unlikely to directly block SPARC activity through a classical ligand-binding mechanism. Instead, this interaction may reflect non-competitive or allosteric modulation, or may represent a structurally permissive but functionally limited association. Importantly, the physiological relevance of this interaction should be interpreted with caution. Therapeutic plasma concentrations of aspirin in humans are typically in the low micromolar range, which may be lower than the concentrations required to achieve meaningful binding to SPARC based on the docking results. Therefore, the observed inhibitory effects of aspirin on SPARC expression and macrophage-like VSMC transformation in our study are more likely mediated through indirect mechanisms, such as modulation of inflammatory signaling pathways or oxidative stress, rather than direct binding alone. Further experimental validation, including quantitative binding assays and structure–function studies, will be necessary to determine whether SPARC represents a direct pharmacological target of aspirin.

## Conclusion

In summary, we found that SPARC overexpression strongly correlated with macrophage-like VSMC biomarkers, which was driven by increased ROS via NRF1 activation. ROS inhibition reduced this macrophage-like transformation, disrupting a vicious cycle of inflammatory signaling and macrophage recruitment. Aspirin was shown to inhibit SPARC expression and attenuate macrophage-like phenotype transformation of VSMCs; however, whether this effect is mediated through direct interaction with SPARC or indirect regulatory mechanisms requires further investigation.

## Data Availability

The original contributions presented in the study are included in the article/supplementary material, further inquiries can be directed to the corresponding authors.
